# The sustained effect of texture-based eating rate on food intake in an 11-d randomised controlled trial

**DOI:** 10.1017/S0007114525106193

**Published:** 2026-05-14

**Authors:** Marieke van Bruinessen, Lise A. J. Heuven, Markus Stieger, Marlou P. Lasschuijt, Ciarán G. Forde

**Affiliations:** 1 Sensory Science and Eating Behaviour Chair, Division of Human Nutrition and Health, Wageningen University & Research, P.O. Box 17, 6700 AA Wageningen, The Netherlands; 2 Food Quality and Design Group, Wageningen University & Research, P.O. Box 17, 6700 AA Wageningen, The Netherlands

**Keywords:** Eating behaviour, Eating rate, Food texture, Food intake, Energy intake

## Abstract

Food texture influences eating rate (ER), and slower ERs are associated with reduced energy intake within a meal. However, it remains unclear whether this acute effect of ER on intake is sustained over time. We investigated whether texture-based differences in meal ER can have a sustained effect on food and energy intake across 11 consecutive days. In a randomised cross-over feeding trial, Dutch adults (*n* 20) were randomised to an 11-d ‘fast’ and an 11-d ‘slow’ ER diet, followed by a 17-d washout period before completing the alternate diet-arm. Participants consumed *ad libitum* breakfast and dinners of which ER was manipulated using food texture and received the same lunch meals on both diets served in regular-sized fixed portions. Diets were matched for served total weight (gram), energy (kcal) and energy density (kcal/gram) and were equivalent for visual volume, meal liking and meal variety. Meal ER on the ‘slow diet’ was on average 32 % slower compared with the ‘fast diet’ (*P* < 0·01). On days when texture led to significant differences in ER, food intake was reduced by 121 (se 24) g/d (*P* < 0·001), and this effect did not attenuate over time (*P* = 0·25). Cumulative food intake was 6 % lower for the slow compared with the fast diet (*P* < 0·001) with no significant difference in energy intake. On 8 of the 11 test days, meal texture reduced ER and supported a consistent reduction in food intake. Further research should test whether a whole diet approach to lowering ER by modifying meal textures could help to moderate food and energy intakes.

Food sensory properties such as sight and smell affect what we choose to eat, whereas a foods’ textural and taste properties affect our eating behaviours, meal size and energy intake^(1)^. During mastication, foods undergo structural changes, particle size reduction and increased lubrication through saliva incorporation to form a safe-to-swallow bolus and the rate of these changes inform our eating rate (ER)^(2)^. ER (g/min) is defined as the amount of food consumed per unit time and is largely influenced by a food’s textural properties. Foods that are harder, drier or that have more elastic or chewier textures, require a longer chewing time to reduce their structure and enhance lubrication of the bolus to be safely swallowed. This results in food spending a longer time in the oral cavity during mastication (longer oro-sensory exposure time) and producing a slower ER^(3–9)^. Previous research has demonstrated that foods that are consumed with a slower ER are eaten in smaller amounts compared to foods consumed with a faster ER^(7–10)^. Previous research suggests an estimated 20 % reduction in meal ER (g/min) will decrease food intake (g) by between 10–15 % and reduce energy consumption by up to 30 % within a meal depending on the energy density of the meal components^(9,11)^.

Slower ERs have been shown to effectively reduce *ad libitum* energy intake within meals^(6–8,12,13)^ and daily energy intake^(14)^ and may have a sustained effect on eating behaviour and a cumulative effect on food intake. In an inpatient randomised controlled trial, participants were randomised to a 14-d ultra-processed (UPF) and to a 14-d minimally processed diet where all meals were served *ad libitum*. Results showed a sustained net difference in average daily energy intake of 508 kcal/d. Participants consumed the UPF diet with a faster ER, and increased their intake of higher energy dense components to yield a 50 % higher energy intake rate (48 *v*. 31 kcal/min), suggesting that ER and energy density may have played a role in the observed differences in energy intake^(15)^. A subsequent study compared *ad libitum* intake for UPF and minimally processed meals that differed in texture-based ER and found that manipulating meal texture led to a 35 % slower ER and this resulted in a 26 % lower energy intake compared to meal textures that promoted faster ER, and this was the same across minimally and UPF meals^(12)^. A follow-up study replicated this result, where meal textures moderated energy intake across UPF and minimally processed meals served for breakfast, lunch and dinner leading to a 21 % reduction in food and a 33 % reduction in energy intake for the meals that were consumed with a slower ER^(14)^. These findings suggest that previously reported differences in energy intake from UPF’s are a product of texture-based differences in ER, and highlight that selecting foods with specific textures to slow down ER could reduce food and energy intake for different meals regardless of their degree of industrial processing. Whether food texture can have a sustained effect on lowering ER and reduce food and energy intake over consecutive days remains unknown.

The current study investigated whether texture-based differences in meal ER can have a sustained effect on food and energy intake across 11 consecutive days. We hypothesise that texture-based differences in ER are stable over time and have a consistent impact on *ad libitum* food (g/d) and energy (kcal/d) intake such that intake is reduced on a slow ER diet compared to a fast ER diet, and that differences in intake are relative to the size of observed differences in ER.

## Methods

### Study design

**Figure 1.**
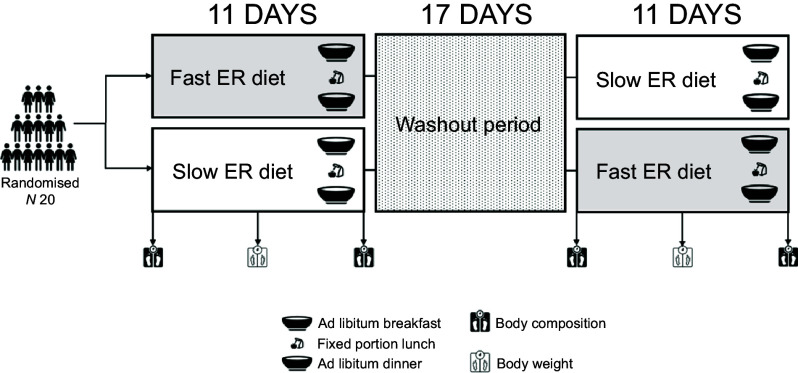
Design of the study. Participants (*n* 20) were randomly assigned to either the fast or slow diet, which consisted of *ad libitum* breakfast (*n* 22 meals) and dinner (*n* 22 meals) with either a texture-based fast or slow eating rates (g/min) and a fixed portion of lunch that was the same for both study diets. After a 17-d washout period, participant received the alternate diet.

The study had a single-blind randomised cross-over design in which participants were initially randomised to an 11-d diet consisting of foods with a fast ER or an 11-d diet consisting of foods with a relatively slower ER. After a 17-d washout period, participants then completed 11 d on the alternate diet arm (Figure 1). On day 12, participants finished their intervention period after breakfast and were allowed to resume their habitual dietary behaviour. The primary outcome of the study was between diet differences in *ad libitum* food intake (g/d) and energy intake (kcal/d) across 11 d. The semi-residential study was performed at the Health Research Unit of Wageningen University and Research, the Netherlands. This study was conducted according to the guidelines laid down in the Declaration of Helsinki and was approved by the Research Ethical Committee of Wageningen University (2022-100-Heuven). The study was registered at Clinicaltrials.gov Clinical Trial registry: NCT05561426 (https://clinicaltrials.gov/study/ NCT05561426). Written informed consent was obtained from all participants.

### Participants

A power calculation (G * Power, version 3.1.9.4 for Windows) indicated that a sample size of *n* 20 provides 80 % power (*α* = 0·05, two-tailed) to detect an average difference of 200 (sd 300) kcal/d (approximately 10 %) in daily energy intake across 11 d, between the two study diets. The effect size was estimated based on previous research^(9,12,13)^.

Dutch participants (18–55 years, BMI: 18·5–30 kg/m^2^) were recruited from Wageningen and surroundings using flyers, posters, and social media. Prospective participants needed to be healthy (self-report), commonly consume three meals per day around the same times at least 5 d a week and understand and speak English. Exclusion criteria included use of medication that could influence study outcomes, food allergies or intolerances for any of the study foods, disliking > 20 % of the study meals (≥ 6 on a 9-point Likert scale), or being a high-restrained eater according to the Dutch Eating Behaviour Questionnaire (DEBQ): cut off for men > 2·89, cut off for women > 3·39^(16)^. Participants (*n* 45) were invited for a screening visit, which included body weight (SECA 704 scale) and height (SECA 213 stadiometer) measurements. Participants were screened for their habitual eating speed and asked to consume a fixed quantity of a carrot stick (15 g) as described previously^(17)^, and were excluded if they demonstrated extreme fast or slow ERs (> 2 × sd from the mean) based on data collected in a previous study^(18)^. Participants were not informed about the objectives of the study, instead they were informed that the aim of the study was to investigate the effect of emotions on eating behaviour. Participants were debriefed when the study was completed, and a post-trial manipulation check revealed that only one participant guessed the true aim of the study correctly. A total of 20 participants (10 male) completed the study and were included for data analysis.

### Study diets

The breakfast and dinner meals were matched for served total weight (gram), energy (kcal), energy density (kcal/gram), energy from ingredients classified as Nova 4 (UPF)^(19)^, and were equivalent for visual volume, meal-liking and variety (i.e. total number of meal components). The diets were matched as closely as possible on macronutrient content, sodium and fibre, but priority was given to the matching of the previously mentioned variables. The energy and nutrient content of the average 11-d diets were calculated from nutrition information presented on food packaging and can be found in Table 1.

**Table 1.** Average diet composition served per day (breakfast, lunch and dinner) for the fast and slow diet

Per day	Fast	Slow
Portion size (g)	2750	2710
Total energy (kcal)	4532	4496
Served energy density (kcal/g)	1·65	1·66
Consumed energy density (kcal/g)	1·52	1·58
Fat (g)	170	157
Carbohydrates (g)	565	574
Sugar (g)	179	182
Protein (g)	161	164
Fibre (g)	57	58
Ultra-processed^*^ (% calories)	97	89

* Nova 4.

The study diets consisted of commercially available food products prepared in a domestic kitchen and meals have been pilot-tested previously^(13)^. For the fast and slow diets, nine breakfasts and dinners were developed consisting of a variety of foods with food textures that required either more (slower ER) or less (faster ER) extensive oral processing. Lunch meals were served as standardised portions and were identical for both study diets (i.e. no ER manipulation). On the weekend, two of the 9-d menus were repeated, creating in total 11 d of intervention (breakfast and dinner), which were block randomised (*n* 5 participants) within the treatment arms (fast and slow). The description and pictures of the menus can be found in the online Supplementary Table 1.

Both diets consisted of food items that would be classified as ‘ultra-processed’ based on the Nova classification system^(19)^, which enabled us to test the impact of ER on energy intake from UPFs and draw comparisons with previous findings^(12–15)^. The Nova system classifies food products into four categories: (1) unprocessed or minimally processed, (2) processed culinary ingredients, (3) processed foods, and (4) UPFs. Nova group 4 defines UPFs as: ‘Formulations of ingredients, mostly of exclusive industrial use, that result from a series of industrial processes’^(19)^. Individual food products were independently classified according to the Nova classification by two researchers familiar with the Nova definitions. Post-hoc comparison showed an average of 93 % agreement between the two researchers for each foods’ Nova category assignment. The remaining 7 % of food products were re-classified by a third researcher to reach 100 % agreement.

### Food and energy intake

In the week prior to the first intervention period, and during the washout period, participants were asked to record their food intake using the validated online food diary application Traqq (Division of Human Nutrition and Health, Wageningen University, The Netherlands) on two weekdays and one weekend day^(20,21)^. Participants’ habitual food and energy intake was estimated using nutrition calculation software based on the food composition table of the Netherlands (NEVO) (Compleat© 2010–2023 Human Nutrition, Wageningen University). During the 11-d intervention periods, all foods were provided by the research team and participants were instructed to only consume the foods provided in the study, with the exception of water, coffee, or tea without sugar or milk. Participants were also instructed to avoid high-intensity exercise and to abstain from consuming alcoholic beverages and drugs.

On weekdays, breakfast and dinner meals were consumed at the research location. In the morning before breakfast, participants arrived in a fasted state. Participants received *ad libitum* breakfast and dinner, which was 200–300 % of a regular portion size for each meal and were instructed to eat in their normal way and eat until comfortably full. Participants received approximately ∼1200 g of food *per* breakfast and dinner meal, and were provided with a new portion if they completed the first one. All meals were served with a glass of water (120 ml) and an additional cup of coffee or tea (140 ml) was offered with breakfast. Participants were offered an initial 25 min to consume their meal, and participants could request to eat for longer if needed. Each meal session lasted an average of 30 min, and participants were obliged to stay for the full session.

All plates, bowls and glasses were covertly weighed before and after meal consumption to determine food and water intake. Meal components were weighed individually on a digital scale with an accuracy of 0·1 g (Denver Instrument, S-4001 Scale Balance). Energy intake was then calculated as a product of the weight consumed of each component and the energy density based on package information. When nutrient information was unavailable, the NEVO table was used for specific food items.

On weekends, all food was prepared centrally at the Health Research Unit at the Division of Human Nutrition, and participants received pre-cooked and pre-packed *ad libitum* portions of their meals, together with easy-to-follow instructions on how to re-heat, consume and collect the leftover foods and packages. Participants were asked to photograph their meal before and after consumption to check if they correctly prepared the weekend meals, and all leftovers were returned to the research facility on Monday for weighing by the research team.

### Assessment of eating rate and eating behaviour characteristics

Eating behaviour characteristics were quantified using behavioural coding analysis of video recordings from each test meal. During the test sessions a webcam (Logitech 640 × 280 pixels) and Action camera (EKEN, H9R Action camera) were positioned in front of the participant at face level, and participants were instructed to limit excessive head movements and look forward as much as possible when eating, though they could not see themselves during recording. Eating behaviours of interest including the number of bites, number of chews, number of swallows, chewing duration (s), oro-sensory exposure duration (s) and total meal duration (min) were quantified using a coding scheme developed previously^(22)^ using the software ELAN Version 6.0 (Max Planck Institute for Psycholinguistics, The Language Archive, Nijmegen, The Netherlands). These behaviours were later used in combination with amount of food (g) consumed to derive average ER (g/min), average bite size (g/bite) and chews per gram (chews/g).

To obtain an estimate of eating behaviours for each test meal in the fast and slow diet arms, the breakfast and dinner meals, a subset (*n* 12) of participants was fully annotated by trained coders. A total of 429 (60 %, twelve participants × thirty-six meals except for three missing videos) of the 720 videos were manually annotated. To assess the consistency between different video annotators (*n* 3 coders for breakfast and *n* 2 coders for dinner meals), re-coded a subset (8 %) of videos. Intra-class correlation coefficients were 0·83–1·00 across all measured eating behaviour characteristics for breakfast and dinner meals, indicating excellent consistency between coders^(23)^ (online Supplementary Table 2).

Average ER (g/min) for each test meal was calculated by dividing the amount eaten (g) by the timed meal duration (min) using the time stamp in the data collection software used to capture participants responses before, during and after each meal (EyeQuestion®, version 5.10.7, the Netherlands). ER obtained from both the timer and software timestamp showed excellent agreement (intra-class correlation coefficients = 0·95); therefore, only ER obtained from the timer are reported in the results from the current study.

### Appetite and liking ratings

Liking of the first bite of the meal was assessed using a 100 mm visual analogue scale anchored by ‘Not at all’ (0) and ‘Extremely’ (100). Familiarity was measured using a nine-point Likert scale. Before and after consumption of the meal, participants rated levels of hunger, fullness, thirst, desire to eat and their prospective consumption on a 100 mm visual analogue scale anchored by ‘Not at all’ (0) to ‘Extremely’ (100). After the meal, participants reported their reason to stop eating based on the Rise-Q-15^(24)^.

### Body composition and physical activity

To assess body weight changes in response to the diet, body weight was measured in duplicate in the morning before breakfast on days 1, 5 and 12 of each intervention period (SECA 704 column scale). In addition, body composition was measured on the first and last morning of each intervention period before breakfast (Day 1 and Day 12). Fat mass and body fat percentage were measured using bio-electrical impedance with a Fresenius Medical Care Body Composition Monitor. Hip and waist circumference were assessed in duplicate using a SECA Measuring tape 201 cm, and waist to hip ratio was calculated by dividing the mean waist value by the mean hip value. During both intervention periods, participants wore an accelerometer (Actigraph GT3X+) on their hip on the right or left side to measure their level of activity. The accelerometer measures were used to estimate participants’ active energy expenditure (kcal/d) and ensure they complied with the request to avoid strenuous physical activity.

### Statistical analysis

All statistical analyses were performed using SAS (version 9.4; SAS Institute Inc.), and *P* values ≤ 0·05 were considered statistically significant. Descriptive statistics are presented as means (se), unless otherwise stated. Prior to data analysis, normality and outliers were visually inspected using QQ-plots, and none of the outcomes were non-normally distributed. Primary outcomes were tested for order and block effects, and no order and block effects were found for daily food and energy intake. Analysis was conducted on an intention-to-treat basis, which included all data available for all participants. Outcome variables that were not measured were treated as missing data and were not modelled.

Repeated measures mixed models (PROC MIXED, SAS) with covariance structure compound symmetry were used to test effects of fixed factors (diet and study day) and their interaction on the primary outcomes: food intake (g/d) and energy intake (kcal/d) on the level of the day and the other outcomes: ER, other eating behaviour characteristics, food and energy intake on the level of the meal and diet, macronutrient intake per day, meal liking and familiarity, pre- and post-meal appetite ratings and changes in body weight and composition. If significant, *post hoc* Tukey tests were conducted to assess effect sizes (difference between the two study arms) and significance. Inclusion of treatment order and block as covariate did not significantly affect daily food or energy intake, suggesting that these are not confounding factors of our study. To determine whether there was a sustained effect of ER on intake independent of the size of the difference in ER (difference between fast and slow), the coefficient of variation of ER was added as a covariate in the same model we used to determine the primary outcomes (food and energy intake). To identify associations between outcome variables ER and food intake, Pearson partial correlations were used controlling for within participant observations (dependency).

## Results

### Participant characteristics

General characteristics of participants are presented in Table 2. Participants (*n* 20, 10 male) were on average 25·3 (sd 5·5) years of age and had a BMI of 23·3 (sd 2·9) kg/m^2^ (range: 18·5–30 kg/m^2^).

**Table 2.** Participant characteristics

	Men (*n* 10)		Women (*n* 10)		Total (*n* 20)	
	Mean	sd	Mean	sd	Mean	sd
Age, years	23·1	2·5	27·5	6·9	25·3	5·5
Body weight, kg	73·3	9·3	73·4	8·2	73·4	9·3
BMI, kg/m^2^	22·8	3·3	23·7	2·6	23·3	2·9
Restrained eater score*	2·0	0·7	2·1	0·5	2·1	0·6

Data are presented as mean (sd).

*Measured with the Dutch Eating Behaviour Questionnaire (DEBQ): cut-off for men > 2·89, cut-off for women > 3·39^(16)^.

### Liking and appetite

Average liking ratings for the test meals were all within the 60–70 range on a 100 mm visual analogue scale, and therefore considered generally ‘liked’ and within the hedonically acceptable range^(25)^. Liking ratings were slightly higher for the slow compared with the fast breakfast meals (*P* < 0·001). Dinner meals of the fast and slow diets were equally liked (*P* = 0·20). Small differences were found in pre- and post-meal appetite ratings (Table 3), but these differences were considered negligible (< 10 mm)^(26)^.

**Table 3.** Liking (100 mm line scale) and familiarity (9-point category scale) of the first bite of the meals and appetite ratings (100 mm line scale) for breakfast and dinner meals assessed before and after consumption of the meals for the fast and slow diet

	Breakfast					Dinner				
	Fast		Slow			Fast		Slow		
	Mean	se	Mean	se	*P* value	Mean	se	Mean	se	*P* value
Liking	61*	2	70^†^	2	< 0·001	68	2	66	2	0·20
Familiarity	3·5*	0·2	3·2^†^	0·2	0·04	3·3	0·2	3·4	0·2	0·49
Hunger										
Pre-meal	62*	3	68^†^	3	< 0·001	68*	2	73^†^	2	< 0·001
Post-meal	14*	1	12^†^	1	0·01	12*	2	15^†^	2	0·02
Δ (post – pre)	–48*	3	–57^†^	3	< 0·001	–56*	3	–59^†^	3	0·05
Fullness										
Pre-meal	21*	2	16^†^	2	< 0·001	21	2	19	2	0·07
Post-meal	70*	3	73^†^	3	0·02	74	3	72	3	0·25
Δ (post – pre)	49*	3	57^†^	3	< 0·001	53	3	53	3	0·71
Thirst										
Pre-meal	62	2	63	2	0·54	54	3	53	3	0·62
Post-meal	28*	3	33^†^	3	0·002	44	3	46	3	0·21
Δ (post – pre)	–34*	3	–29^†^	3	0·03	–10	4	–7	4	0·17
Desire to eat										
Pre-meal	65*	3	71^†^	3	< 0·001	71	2	72	2	0·15
Post-meal	15	2	15	2	0·54	16*	2	19^†^	2	0·02
Δ (post – pre)	–50*	3	–56^†^	3	< 0·001	–54	4	–53	4	0·40
Prospective consumption										
Pre-meal	65*	3	67^†^	3	0·03	69	3	71	3	0·05
Post-meal	23*	2	21^†^	2	0·02	21*	2	23^†^	2	0·03
Δ (post – pre)	–42*	4	–47^†^	4	0·002	–49	4	–48	4	0·53

*Data are presented as mean (se).

^†^Means in a row without a common superscript letter differ *P* < 0·05 with Tukey adjustments for multiple comparisons.

The most selected response on the reasons to stop eating questionnaire was ‘I was full’ (60 %) for both breakfast and dinner, confirming that participants self-reported that they ate until comfortably full. The next most common reason was ‘the food is no longer appealing to me’ (11·7 %). All reasons for why participants stopped eating are summarised in online Supplementary Table 3.

### Eating rate and characterisation of eating behaviour

**Figure 2.**
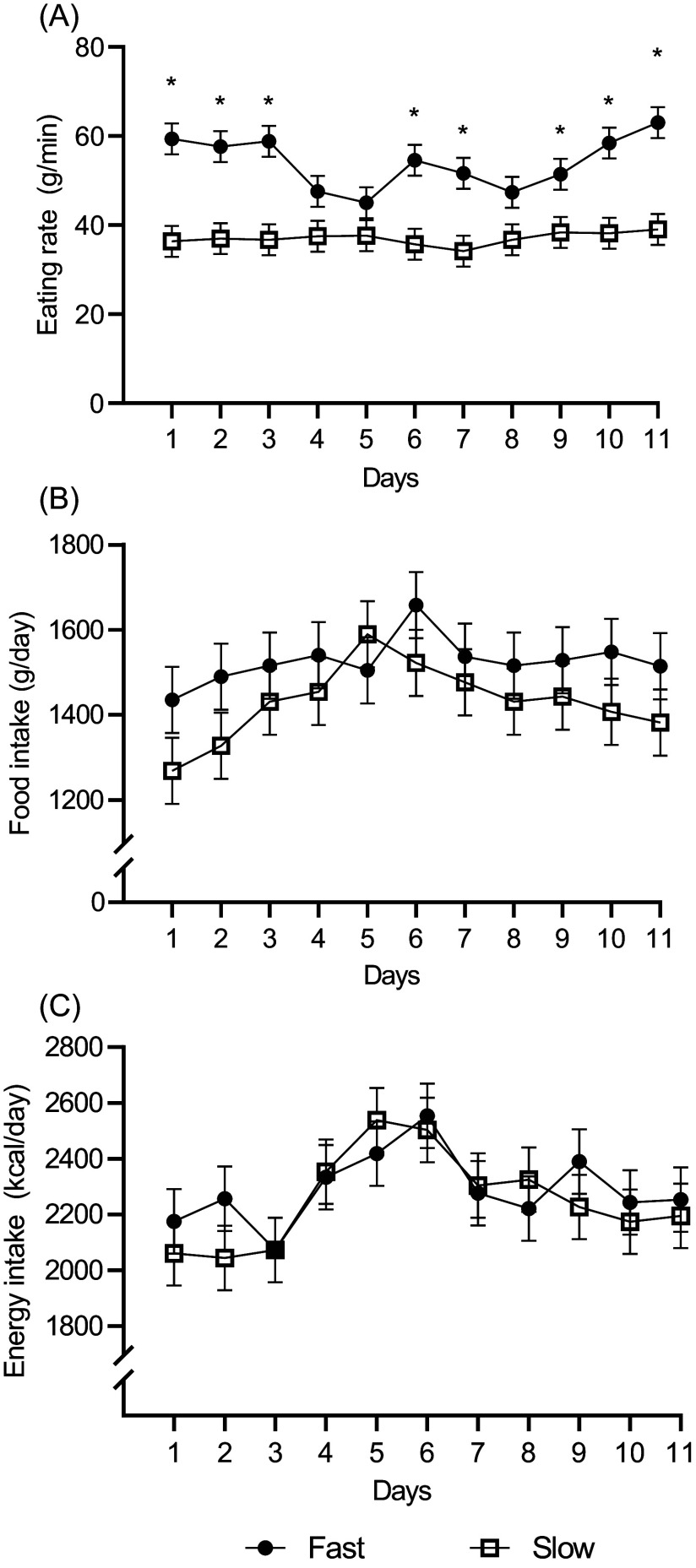
Figure 2 long description. Eating rate (a), daily food intake in grams (b) and daily energy intake in kcal (c) per study day across the 11-d intervention period for the fast and slow diets. Values are mean, *n* 20, repeated- measures mixed models. *P < 0·05 *post hoc* Tukey test.

Meal ER was significantly lower on the slow diet compared with the fast diet on 8 of the 11 test days (main effect diet: *P* < 0·001). The coefficient of variation of ER significantly differed between the two diets (fast: 25 (se 1), slow: 18 (se 1), *P* < 0·001), indicating a wider variation in ER across the 11-d period for the fast diet compared with the slow diet (Figure 2(a)). Overall, meal ER (g/min) was on average 32 % lower for the slow compared with the fast diet, with an average 11 (se 2) g/min (20 %) and 23 (se 1) g/min (40 %) lower ER for the slow compared with the fast breakfast and dinner meals, respectively (*P* < 0·001). Meals on the slow diet were consumed with an average 7 (se 2) g smaller bite size (*P* = 0·009), more chews *per* gram (*P* < 0·001) and 3 (se 0·4) min longer total meal duration (*P* < 0·001) compared with meals on the fast diet. Mean eating behaviour characteristics for the breakfast and dinner meals are summarised in Table 4.

**Table 4.** Eating behaviour characteristics for the breakfast and dinner meals per study diet Table 4 long description.

	Diet				ANOVA Fixed Effect *P* value		
	Fast		Slow				
	Mean	se	Mean	se	Diet	Day	Diet × Day
Breakfast							
Eating rate (g/min)	51^†^	4	40^‡^	4	< 0·001	0·09	0·03
Energy intake rate (kcal/min)	79^†^	4	62^‡^	4	< 0·001	< 0·001	0·02
Total meal duration (min)^*^	9^†^	0·5	11^‡^	0·5	< 0·001	< 0·001	0·02
Total OSE time (s)^*^	380^†^	35	552^‡^	35	< 0·001	0·001	0·02
OSE per bite (s/bite)^*^	16^†^	1	13^‡^	1	< 0·01	0·05	0·05
Chews per bite^*^	20^†^	1	16^‡^	1	0·02	0·1	0·04
Bite size (g)^*^	21^†^	3	11^‡^	3	0·03	0·45	0·39
Dinner							
Eating rate (g/min)	57^†^	3	34^‡^	3	< 0·001	0·01	0·12
Energy intake rate (kcal/min)	75^†^	4	50^‡^	4	< 0·001	0·2	0·30
Total meal duration (min)^*^	13^†^	1	18^‡^	1	< 0·001	< 0·001	0·52
Total OSE time (s)^*^	556^†^	45	834^‡^	45	< 0·001	< 0·001	0·63
OSE (s/bite)^*^	12^†^	1	16^‡^	1	< 0·001	0·54	0·18
Chews per bite^*^	15^†^	2	22^‡^	2	< 0·001	0·51	0·41
Bite size (g)^*^	14^†^	0·5	10^‡^	0·5	< 0·001	< 0·001	0·01

* Assessed with video coding of fast breakfasts *n* 118, slow breakfast meals *n* 120, fast dinner meals *n* 95 and slow dinner meals *n* 96.

^†^OSE, oro-sensory exposure.

^‡^Values are means (se). Means in a row without a common superscript letter differ *P* < 0·05 with Tukey adjustments for multiple comparisons.

### Food and energy intake

**Figure 3.**
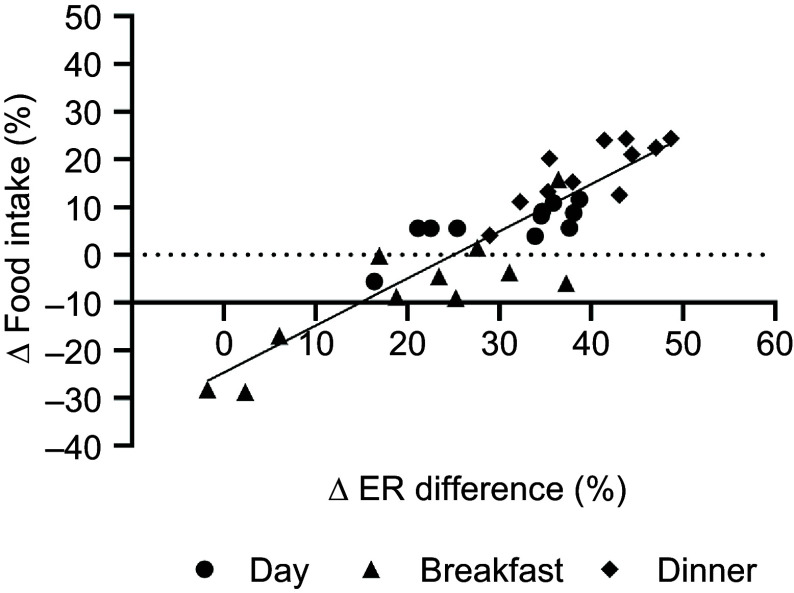
Plot of the effect sizes (fast-slow) of eating rate (ER) and food intake of the fast and slow diets for breakfast and dinner meals and per study day. The black line represents the regression line of the best fit based on average values.

There was a significant reduction in average food intake of 121 (se 24) g/d on 8 out of 11 d where ER was significantly lower on the slow diet compared with the fast diet (main effect diet: *P* < 0·001). Daily food intake was similar in week one and two for both study diets (*P* = 0·25), indicating no significant attenuation of the ER changes or intake over the 11 d of each diet intervention arm, as is shown in Figure 2(b). Cumulative differences in food intake across the 11 d of the diet intervention were 1056 (se 322) g lower on the slow compared with the fast diet (main effect diet: *P* < 0·001). Differences in intake between study conditions were dependent on the difference in ER between the meals of the fast and slow study conditions, such that a 32 % reduction in ER led to a 6 % reduction in total food intake (g). After correcting for the strength of the manipulation of texture-based ER (based on the coefficient of variation), there remained an independent effect of ER on food intake which was sustained over time (*P* < 0·001) (Figure 3).

Observed differences in food intake at the level of the ‘day’ were primarily derived from differences in intake at the evening meal, with 130 (se 14) g lower intake (*P* < 0·001) for the slow, compared with the fast meals. Food intake differences at breakfast showed a reversed trend, and intake was on average higher for the slower compared with the faster breakfast meals (42 (se 13) g, *P* = 0·005). Across both study diets, meal ER was significantly positively correlated with food intake (*r* = 0·56, *P* < 0·001).

Despite matching for dietary energy density, as this was an *ad libitum* paradigm participants were free to eat what they wanted, consumed energy density was on average 0·06 (se 0·02) kcal/g higher for the slow compared with the fast diet (*P* = 0·001) (Table 1). Average daily energy intake did not significantly differ between the study diets (main effect diet: *P* = 0·18) as shown in Figure 2(c) and did not differ from habitual daily energy intake derived from the food diary (*P* = 0·13). Energy intake was on average 56 kcal (8 %) higher (*P* = 0·007) for the slow compared with the fast breakfast meals, whereas it was 84 kcal (9 %) lower (*P* = 0·002) for the slow compared with the fast dinner meals. When correcting for the strength of the manipulation (coefficient of variation), total average daily energy intake was not affected by the diet intervention (*P* = 0·48). Mean food and energy intake per diet and per meal type are summarised in online Supplementary Table 4.

Intake from different macronutrient sources was similar for the fast and slow diets, with no significant differences in daily intake of carbohydrates (*P* = 0·68), sugar (*P* = 0·32), fibre (*P* = 0·95) and protein (*P* = 0·41) (online Supplementary Table 4). Only fat intake was on average 9·9 (se 2·2) g/d higher (*P* < 0·001), and sodium intake was on average 0·5 (se 0·2) g/d higher (*P* = 0·006) on the fast compared with the slow diet.

### Body composition and active energy expenditure

Active energy expenditure was similar for participants on the fast and slow diet (Slow: 396 (se 29) kcal/d and Fast: 381 (se 29) kcal/d, *P* = 0·34) and when added as a covariate did not significantly affect daily food (*P* = 0·29) or energy intake (*P* = 0·19). Participants lost on average 0·7 (se 0·3) kg body weight attributed to a loss in fat mass (main effect day: *P* = 0·006) on both diets, with no significant difference between the two study diets (*P* = 0·32) (online Supplementary Table 5).

## Discussion

The current study tested whether there is a sustained effect of meal textures on ER and food intake (g/d) across 11 consecutive days. Our meal texture intervention supported a significant reduction in average ER and food intake on 8 out of 11 intervention days. For the remaining test days, differences in ER were not sufficient to prompt a difference in ER or intake between the two diets, highlighting the consistency of the link between ER and intake. Meals were consumed on average at a 32 % slower ER resulting in an average reduction of 6 % in food intake over the 11 intervention days. Only breakfast and dinner meals were manipulated, with no texture intervention for lunch or snacks. Whereas intake of macronutrients was similar for the two diets, energy density consumed was slightly higher on the slow diet compared with the fast diet and might have offset the differences in the amount of food consumed (g), resulting in equivalent energy intakes between the two diet arms. Body weight and composition did not differ significantly between the fast or slow ER diet interventions. Importantly, meal texture-based reductions in ER did not attenuate over the 11-d intervention period, suggesting that participants do not adapt to the intervention over this time period.

Whereas previous research has explored the impact of food texture and ER on intake within a meal^(8,12,13)^ or over the course of a day^(14)^, the current study demonstrates that meal texture-based reductions in ER are likely to have a sustained effect on ER and *ad libitum* food intake over a longer period. Despite differences in food intake, post-meal appetite sensations were not significantly different between the diets and all meals fell within the acceptable hedonic range. However, on three of the eleven test days, the texture manipulation presented was not sufficient for a reduction in ER and had a negligible impact on intake. Consuming meals at a faster rate has been identified as a modifiable risk factor for weight gain^(27)^ and extensive observational research has shown associations between faster ER, greater abdominal adiposity, higher BMI and an increased risk of obesity^(28)^. In the past, several approaches have been taken to slow eating speed in an effort to reduce meal size and control body weight over time^(29–31)^. For example, efforts have focused on raising awareness of eating speed during meal times and applied devices to retrain a slower eating behaviour using computerised feedback (i.e. the Mandometer)^(29,30)^ or an augmented fork^(31)^ that sends feedback on ER during food consumption. Previous intervention trials have shown significant reductions in both energy intake and body weight through the use of programmes focused on re-training an individual’s ER^(29,32,33)^. There is broad agreement that the widespread availability of energy dense foods that are served in large portions and that can be consumed rapidly contribute to a food environment that promotes higher habitual energy intakes^(34)^. Efforts are underway to control portion size and regulate upper limits of energy density^(35,36)^. Further opportunities exist to reduce eating speed and moderate energy intake through the application of food’s textural properties^(3)^. Combinations of food texture, structural and lubricant properties have been shown to consistently influence oral processing behaviours and ER, across a wide range of everyday foods^(9)^. An advantage of using texture to slow ER and reduce intake is that it is relatively simple to implement, by substituting hedonically equivalent foods and applying naturally occurring variations in food and meal texture to slow consumption. Second, it does not require significant cognitive effort or attention to adhere to the intervention, as consumers naturally adjust their eating behaviours to the texture challenge they are consuming^(9)^. Further research is needed to better understand the optimal combinations of meal textures that can produce a consistent effect on ER and energy intake. The current trial utilised texture manipulations for the breakfast and dinner meals only, but held the lunchtime meal constant for both diet arms. Given the relatively small sample size, these preliminary findings should be further explored in the future with a larger sample population. Additionally, future research is needed to establish whether it is possible to further enhance the effect of slowing ER across all meals and snacks within a diet intervention, and test whether texture-based ER can contribute to a sustained reduction in energy intake and support healthy body weight maintenance over a longer period of time.

The majority of meals tested in the current diet intervention were categorised as ‘ultra-processed’ based on the Nova scheme for both the ‘slow’ (89 %) and ‘fast’ (97 %) diets. Previous findings have confirmed that food texture rather than degree of processing was moderating food and energy intake for both ultra- and minimally processed meals^(12–14)^. We chose not to include a minimally processed arm for comparison in the current trial as serving a diet of > 85 % minimally processed food is a further intervention in addition to the ER manipulation and is not a true control diet as it does not reflect the everyday foods participants typically consume. Findings from the current trial suggest that when texture-based differences in ER are large enough, they can support reductions in intake for UPF^(37)^. These differences can be further accentuated when combined with reductions in energy density^(38)^. Previous research has highlighted that energy intake from UPF is increased when faster ER is combined with higher energy density, that is, when energy intake rate (kcal/min) is increased^(38–40)^. Consumers tend to compensate poorly when meal energy density is covertly manipulated^(41,42)^, and evidence from the current trial highlights the lack of later compensation for reduced intake from slower ER’s, the stability of using meal texture to reduce ER over time and suggests that both approaches could be applied in combination to have a complementary impact on energy intake.

While food intake results are in line with previous research^(7,8,12,14)^, we report no significant difference in daily energy intake between the study diets. Higher energy density has been associated with higher acute energy intakes^(42)^ and is known to have a greater effect on energy intake than ER^(38)^. A limitation of the study is that we matched energy density at the level of the meal, but not for individual meal components, making it possible to select more energy dense meal components within the slower meals, and less energy dense meal components within the faster meals. This led to equivalent energy intakes between the two diet arms despite differences in food intake (g). With equivalent daily energy intakes between the two study diets, there were no differences in body weight or composition at the end of the intervention period. Participants lost a similar amount of body weight (0·7 kg), which may be attributed to the strict dietary guidance and restricting the consumption of alcohol, soft drinks and snacks during the intervention period^(43–46)^.

Predicting ER of a meal or diet *a priori* is challenging, as consumers adjust their oral processing behaviours in an individual way to the textures presented within each meal. The current intervention successfully reduced ER on most test days using meal texture modifications derived from commercially available foods and relied on meal components with different geometric, size, shape, lubrication and texture properties known to differ in ER^(47–55)^. Food intake tracked with the reduction in ER and was not changed on the days when ER was equivalent across the two diets, highlighting the close association between eating speed and intake. Food texture manipulations were more successful for the dinner meals with larger texture-based differences in ER leading to greater differences in food intake compared with the breakfasts. The less pronounced ER effect for the breakfast meals may be due to individual food preferences. In the current study, we aimed to match for meal palatability. Despite extensive a priory testing, there was still a difference in liking for the breakfast meals between the two diets, which may have counteracted the effect of ER and offset food intake, highlighting the difficulty of predicting individual food preferences. There remains a challenge in understanding the optimal texture combinations that will produce less variation in ER and the most consistent effect on intake across a wide variety of meals. Combinations of texture manipulations are likely to have the strongest effect in reducing ER^(56,57)^, with combinations of small changes to meal texture properties effective in reducing ER and intake, while sustaining the hedonic appeal of the meal. Future research is needed to empirically understand how eating behaviours and intake can be moderated by the texture experience within everyday meals and snacks.

### Conclusion

We demonstrated that when texture-based manipulations to moderate ER are effective, daily food intake (g) is reduced and that this effect is consistent over time. It may be possible to reduce food intake through the use of sensory cues and texture-based reductions in ER, and these food properties are likely to contribute to observed differences in *ad libitum* intake for UPF diets. Our findings contribute to a growing body of evidence that show that energy intake from UPF is influenced by texture properties and offer new opportunities for the development of food-based strategies to moderate food intake and promote healthy body weight maintenance.

## Supporting information

van Bruinessen et al. supplementary material van Bruinessen et al. supplementary material

## Supplementary material

For supplementary material/s referred to in this article, please visit https://doi.org/10.1017/S0007114525106193

## Figure 2 Long description

Panel A: A line graph shows the eating rate in grams per minute over 11 days. The x-axis represents days, and the y-axis represents the eating rate. Two lines represent fast and slow diets, with the fast diet showing a consistently higher eating rate. Significant differences are marked with asterisks. Panel B: A line graph depicts daily food intake in grams over 11 days. The x-axis represents days, and the y-axis represents food intake. Two lines represent fast and slow diets, with the fast diet showing higher food intake. Panel C: A line graph illustrates daily energy intake in kilocalories over 11 days. The x-axis represents days, and the y-axis represents energy intake. Two lines represent fast and slow diets, with the fast diet showing higher energy intake.

Navigate back to Figure 2.

## Table 4 Long description

The table presents a comparison of eating behavior characteristics for breakfast and dinner meals across two diets, labeled as Fast and Slow. It includes mean values and standard errors (SE) for various metrics. The table has 11 rows and 8 columns. Column headers are: Diet, Fast Mean, Fast SE, Slow Mean, Slow SE, ANOVA Fixed Effect P value Diet, Day, Diet x Day. Row labels include: Eating rate (g/min), Energy intake rate (kcal/min), Total meal duration (min), Total OSE time (s), OSE per bite (s/bite), Chews per bite, Bite size (g). Each row provides data for both breakfast and dinner. Notable trends include significant differences in eating rate, energy intake rate, total meal duration, total OSE time, OSE per bite, chews per bite, and bite size between the fast and slow diets, with P values indicating statistical significance.

Navigate back to Table 4.
