# Supplementary material for: The sustained effect of texture-based eating rate on food intake in an 11-d randomised controlled trial
Source: Br J Nutr. 2026 Jan 6;135(9):1006–14. doi: 10.1017/S0007114525106193 (PMC13315526; doi:10.1017/S0007114525106193)
Supplement: van Bruinessen et al. supplementary material [file S0007114525106193sup001.docx]

The sustained effect of texture-based eating rate on food intake in an 11-day randomized controlled trial

Marieke van Bruinessen^1^, Lise A.J. Heuven ^1,2^, Markus Stieger^1,2^, Marlou P. Lasschuijt^1^ and Ciarán G. Forde^1*^

^1^ Division of Human Nutrition and Health, Wageningen University & Research, P.O. Box 17, 6700 AA, Wageningen, The Netherlands

^2^ Food Quality and Design group, Wageningen University & Research, P.O. Box 17, 6700 AA, Wageningen, The Netherlands

**Supplemental Table 1** Ingredients and pictures of the menus of the fast and slow diet.

| **Day menu 1** | | |
| --- | --- | --- |
|  | **Fast** | **Slow** |
| **Breakfast** | 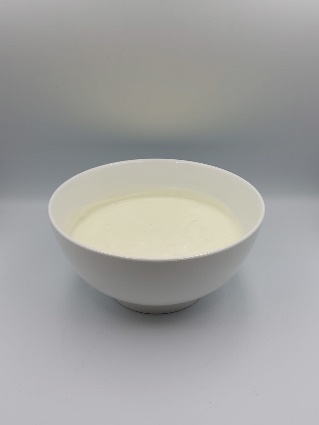 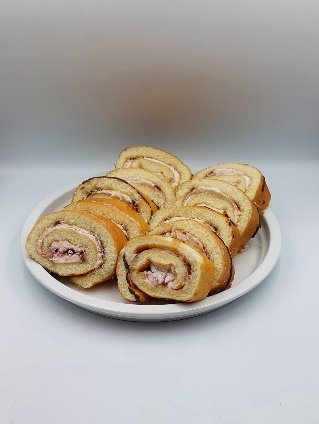 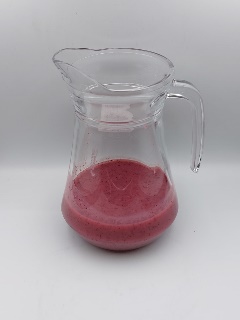 | **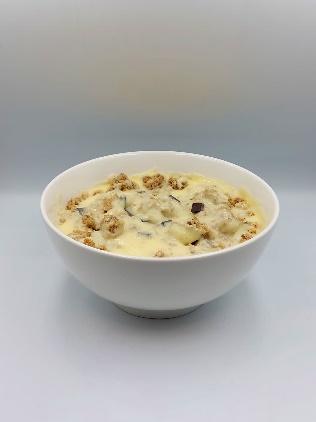 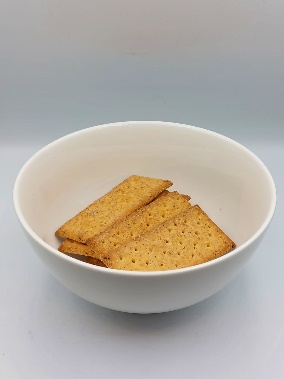 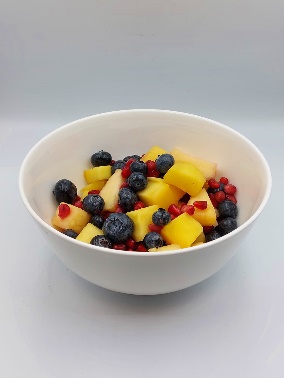** |
|  | - 400 g Vanilla yoghurt (den Eelder) - 430 g cake roll with strawberry cream filling (Albert Heijn) - 375 g forest fruit breakfast smoothie (Coolbest) | - 560 g vanilla custard (de Zaanse Hoeve) with 160 g chocolate cruesli (Quacker) - 93 g savoury biscuits (Sultana) - 390 g pineapple, mango, pomegranate fruitmix (Albert Heijn) |
| **Lunch** | - 164 g ham, cheese egg sandwich (The Bread Office) - 40 g raisin bun (Albert Heijn) - 135 g apple | - 164 g ham, cheese egg sandwich (The Bread Office) - 40 g raisin bun (AH private label) - 135 g apple |
| **Dinner** | 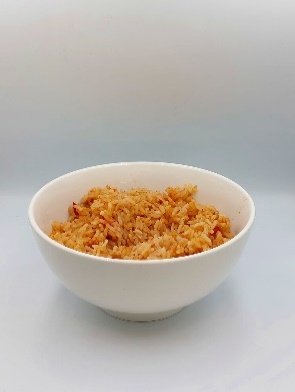 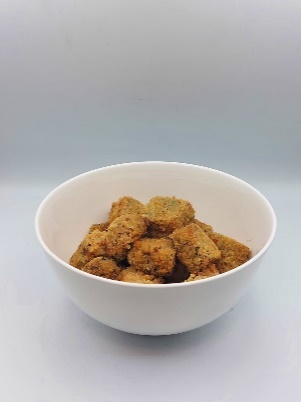 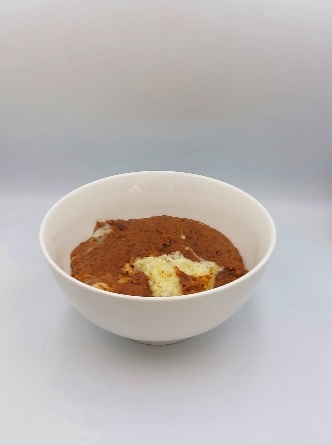 | 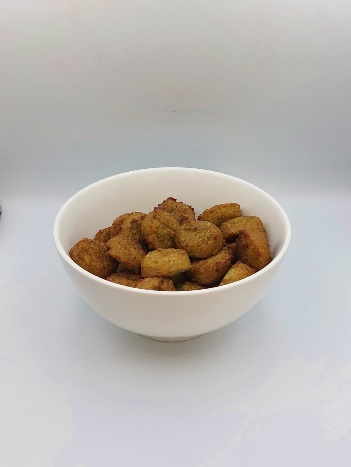 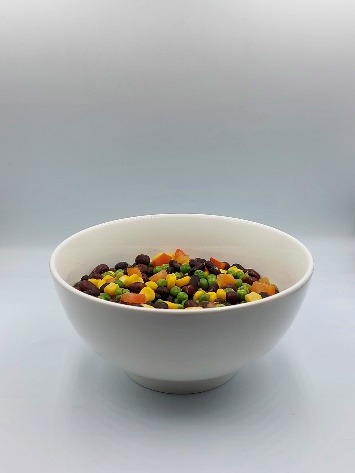 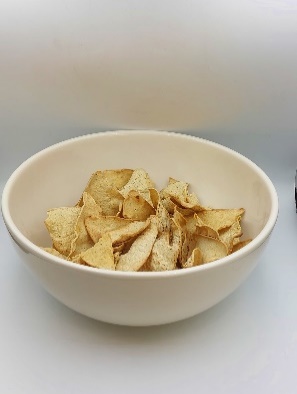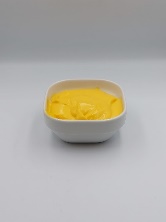 |
|  | - 330 g Hungarian rice (Huuskes private label) with 100 g salsa mild (Albert Heijn) - 360 g carre with sundried tomato (Garden Gourmet) - 350 g refried beans (La Morena) with 60 g grated cheese 30 + (de Zaanse Hoeve) | - 410 g falafel (Albert Heijn) - 438 g Mexican vegetable mix (Iglo) - 150g Totopos chips (La Morena) - 20g Cheese dip (Santa Maria) |
| **Day menu 2** | | |
|  | **Fast** | **Slow** |
| **Breakfast** | 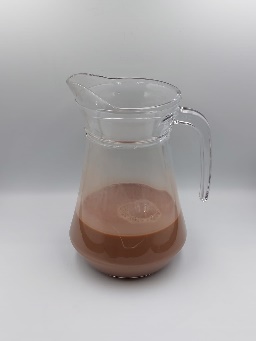 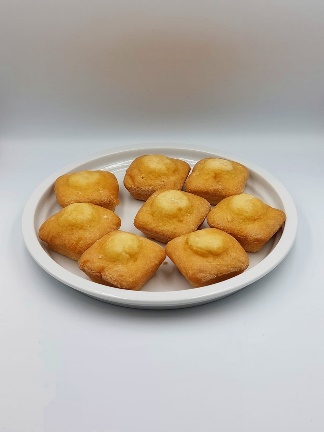 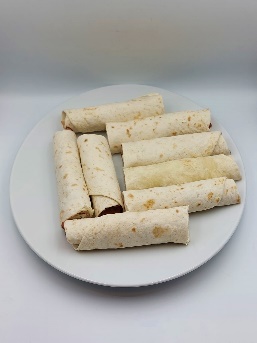 | 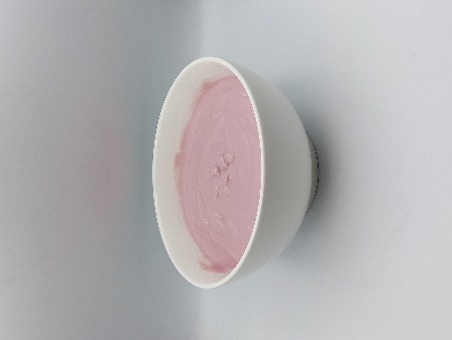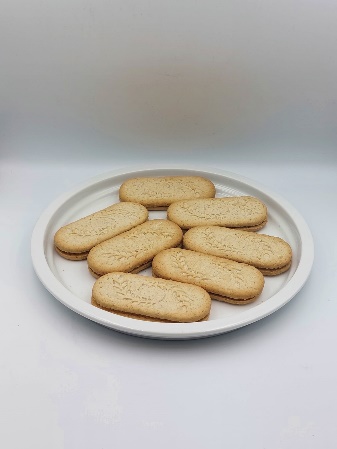 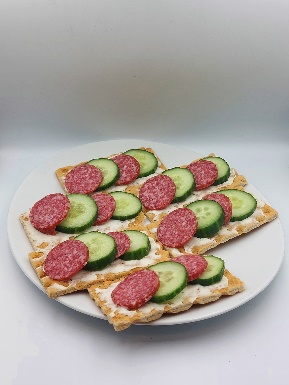 |
|  |  |  |
|  | - 520g Chocolate protein drink (Melkuni) - 224g Lemon cakes (Cereal) - 248g Tortilla wrap (Albert Heijn) with 80g cream cheese with herbs (Garlan) and 130g Grilled chicken sausage | - Plant-based blueberry flavored yoghurt (Oatly) - Sandwich biscuit (Belvita) - Crackers (Wasa) with light cream cheese with herbs (AH private label) with cucumber and salami (Stegeman) |
| **Lunch** | - 79g White bun with ham (The Bread Office) - 79g White bun with cheese (The Bread Office) - 30g Sweet egg bun (Albert Heijn) - 150g Pineapple | - 79g White bun with ham (The Bread Office) - 79g White bun with cheese (The Bread Office) - 30g Sweet egg bun (AH private label) - 150g Pineapple |
| **Dinner** | 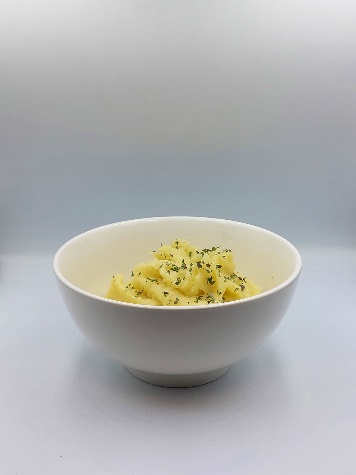 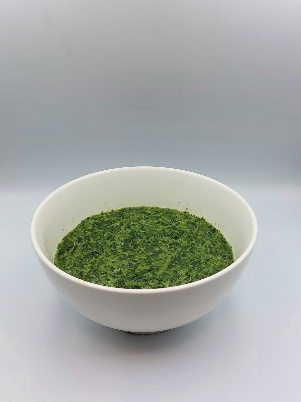 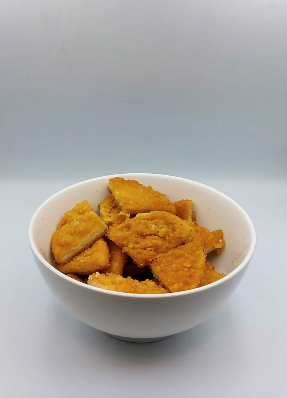 | 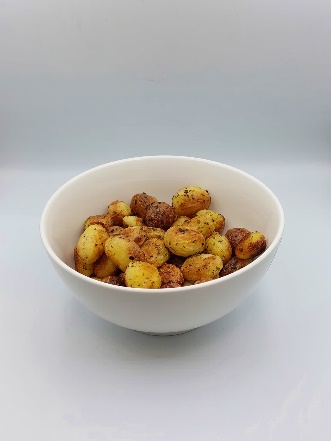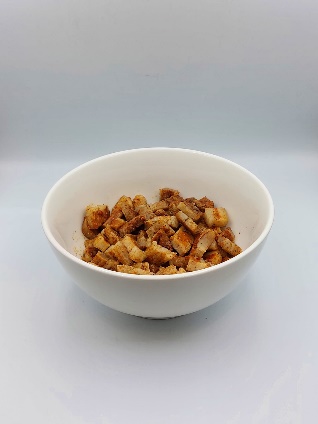 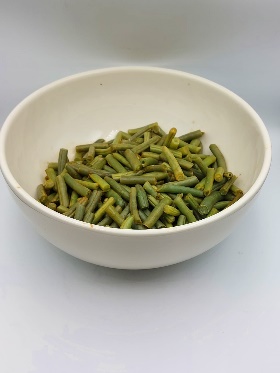 |
|  | - 450g Potato puree (Albert Heijn) with 1g Italian Herb mix (Euroma) - 400g Spinach with cream (Iglo) - 360g Pluimfeestburger (The Vegetarian Butcher) | - 450g Roasted potatoes with herbs (Albert Heijn) - 272g Bacon with herbs (Albert Heijn) - 600g Green beans with Tandoori sauce (Patak) |
| **Day menu 3** | | |
|  | **Fast** | **Slow** |
| **Breakfast** | 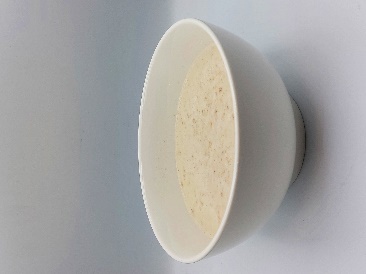 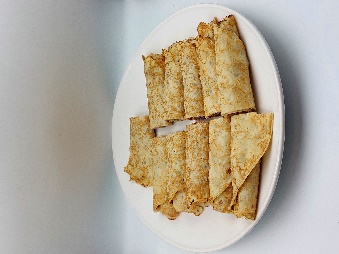 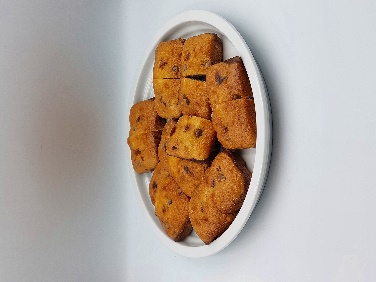 | 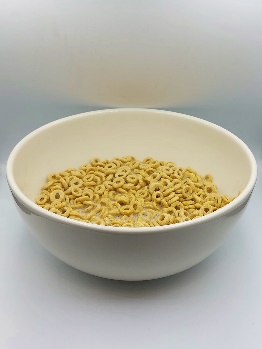 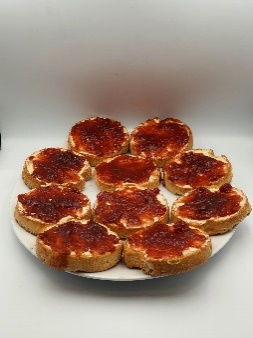 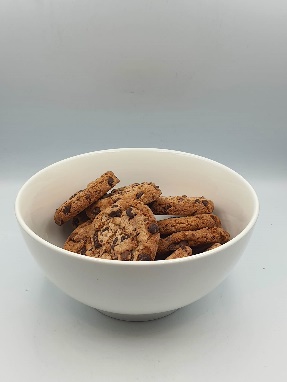 |
|  | - 132g Oats (Quaker) with 360g oatdrink (Oatly) - 371.25g pancake (Jan) with 82.5g strawberry jam (Albert Heijn) - 262.5g Chocolate chip cakes (Milka) | - 170g Honey loops (Kellogs) with 510g Oat drink (Alpro) - 100g Rusks (Albert Heijn) with 50g light margarine (Becel) and 150g Strawbery jam (Albert Heijn) - 225g Chocolate chip cookies (Albert Heijn) |
| **Lunch** | - 157g Roasted chicken sandwich (The Bread Office) - 37.5g Liga evergreen (Liga) - 150g White grapes | - 157g Roasted chicken sandwich (The Bread Office) - 37.5g Liga evergreen (Liga) - 150g White grapes |
| **Dinner** | 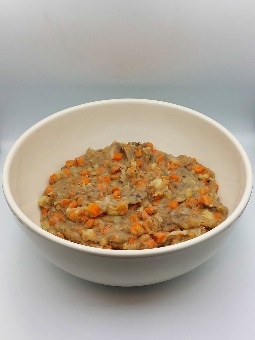 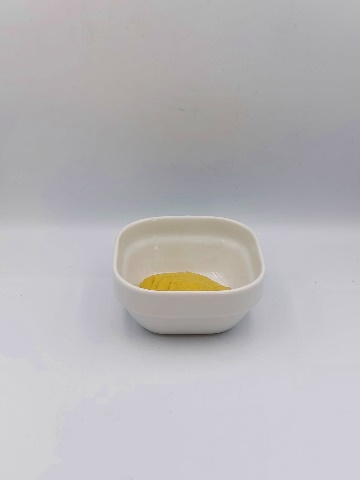 | 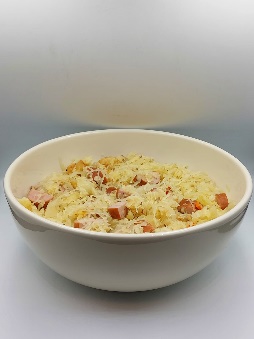 |
|  | - 1160g Hutspot with Hachee (Albert Heijn) - 40g Mustard (Marne) | - 350g small Rösti rounds (Aviko) with 670g sauerkraut with herbs (Albert Heijn) and 230g smoked sausage (Unox) |
| **Day menu 4** | | |
|  | **Fast** | **Slow** |
| **Breakfast** | 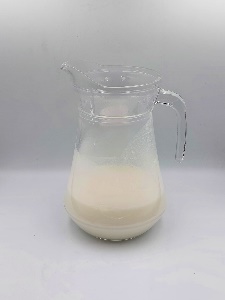 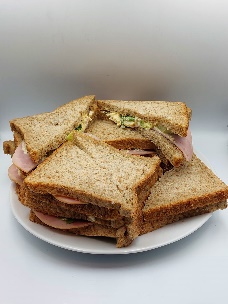 | 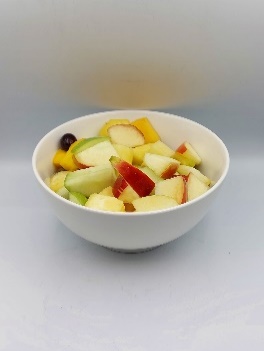 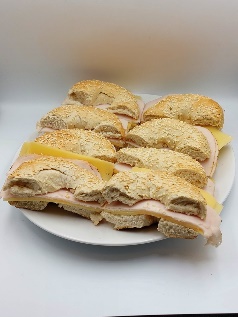 |
|  | - 420g Protein drink vanilla cookie (HiPRO) - 420g Casino volkoren bread (Albert Heijn) with 120g ham (Albert Heijn), 210g egg salad (Johma) and lettuce (Albert Heijn) | - 550g pineapple, mango, apple and grape fruit salad (Albert Heijn) - 340g sesame bagels (Albert Heijn) with 128g chicken (Albert Heijn) and 200g cheese (Milner) |
| **Lunch** | - 157g Bacon egg sandwich (The Bread Office) - 25g B’tween bar peanut butter (Hero) - 160g mandarins | - 157g Bacon egg sandwich (The Bread Office) - 25g B’tween bar peanut butter (Hero) - 160g mandarins |
| **Dinner** | 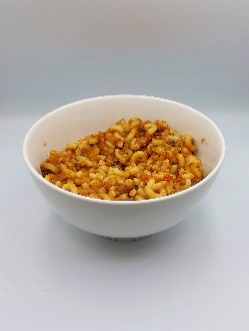 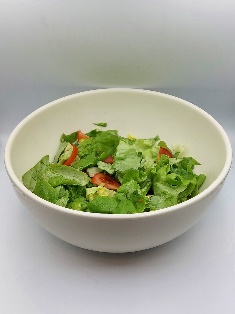 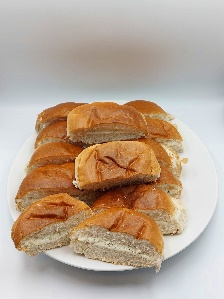 | 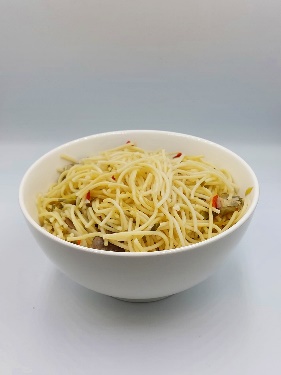 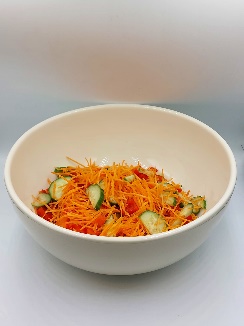 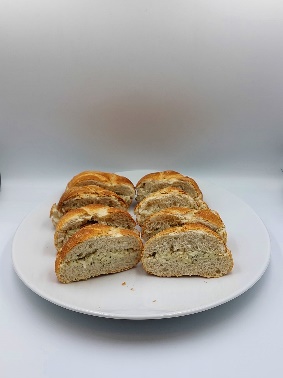 |
|  | - 580g Macaroni Bolognese (Huuskes) - 100g lettuce with 100g tomatoes and 25g natural dressing (Albert Heijn) - 325g white buns (Albert Heijn) with 78g cream cheese herbs (Boursin) | - 580g Spaghetti with vegetables (Huuskes) - 120g red bell pepper, 120g cucumber, 120g carrot julienne (Albert Heijn) and 10g zero calorie dressing (Remia) - 200g Kaiser rolls (Albert Heijn) with 72g herb butter (Albert Heijn) |
| **Day menu 5** | | |
|  | **Fast** | **Slow** |
| **Breakfast** | 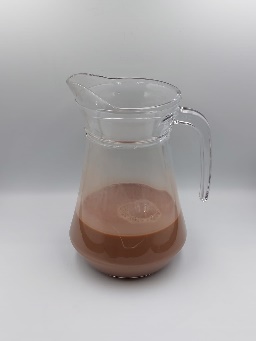 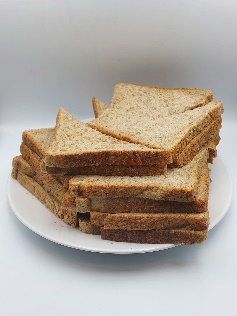 | 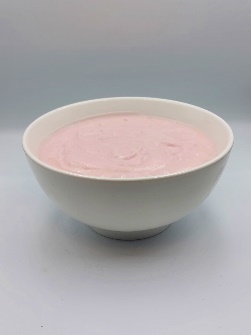 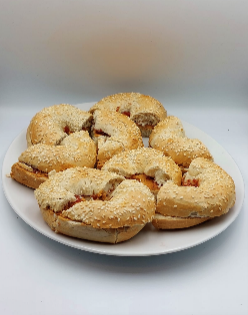 |
|  | - 620g chocolate protein drink (Melkuni) - 455 casino wholegrain bread with 65g peanut butter light (Calvé) and 65g strawberry jam (Albert Heijn) | - 740g strawberry quark - 382.5g sesame bagels (Albert Heijn) with 45g peanut butter (Skippy) and 45g strawberry fruit spread (Zonnatura) |
| **Lunch** | - 157g Roasted chicken sandwich (The Bread Office) - 37.5g Liga evergreen (Liga) - 150g White grapes | - 157g Roasted chicken sandwich (The Bread Office) - 37.5g Liga evergreen (Liga) - 150g White grapes |
| **Dinner** | 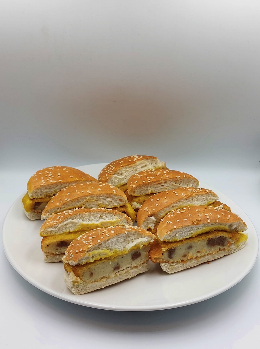 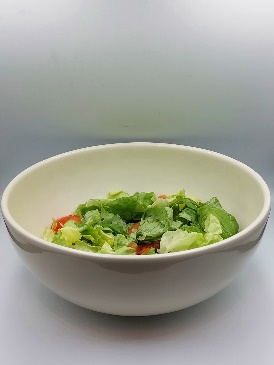 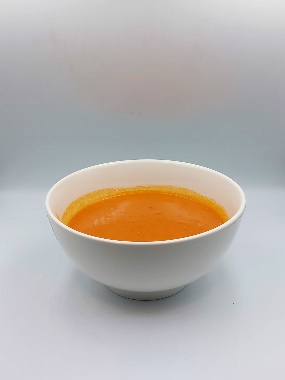 | 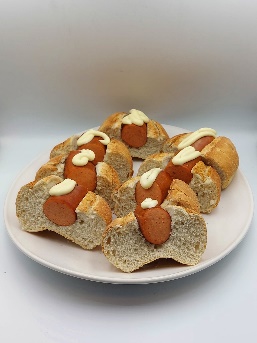 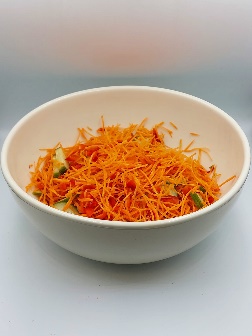 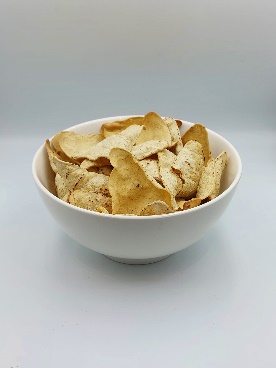 |
|  | - 600g Croquette burger (Mora) - 80g lettuce with 80g tomatoes and 10g zero calorie dressing (Remia) - 440g creamy tomato soup (Unox) with 15g liquid baking butter (Becel) | - 280g white bun (Albert Heijn) with 280g spicy smoked sausage (Unox) and 40g fries sauce (Remia) - 170g red bell pepper, 170g cucumber, 170g carrot julienne (Albert Heijn) and 15g zero calorie dressing (Remia) - 105g Totopos chips (La Morena) |
| **Day menu 6** | | |
|  | **Fast** | **Slow** |
| **Breakfast** | 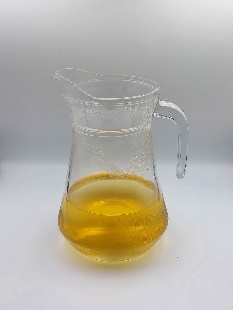 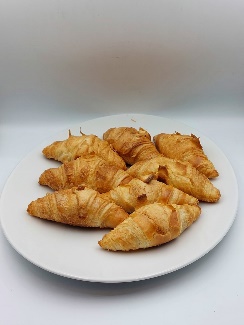 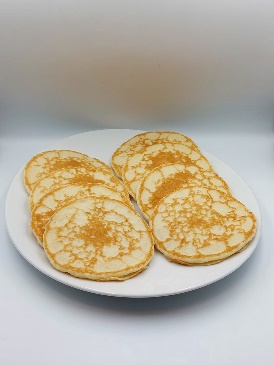 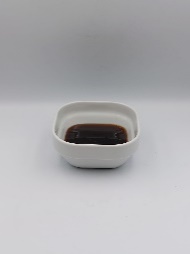 | 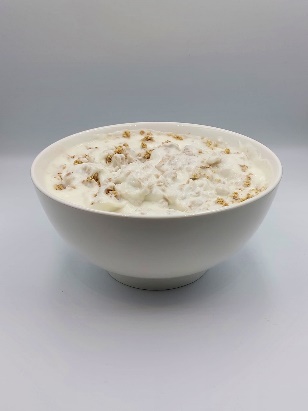 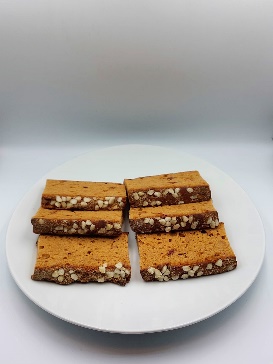 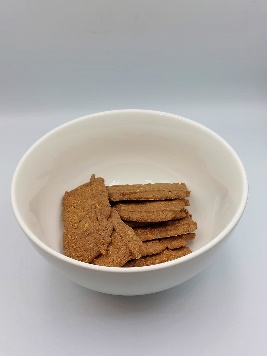 |
|  | - 540g Apple juice (Appelsientje) - 200g mini croissants (Albert Heijn) - 400g pancakes (OaYeah) with 80g syrup | - 742 vanilla quark (Optimel) with 159g cruesli (Albert Heijn) - 214g Gingerbread (Peijnenburg) - 88g spiced biscuits (Céréal) |
| **Lunch** | - 157g Bacon egg sandwich (The Bread Office) - 25g B’tween bar peanut butter (Hero) - 160g mandarins | - 157g Bacon egg sandwich (The Bread Office) - 25g B’tween bar peanut butter (Hero) - 160g mandarins |
| **Dinner** | 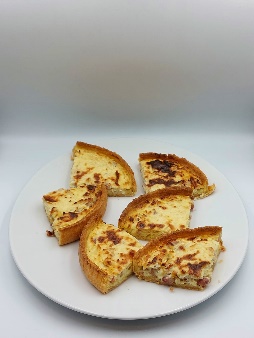 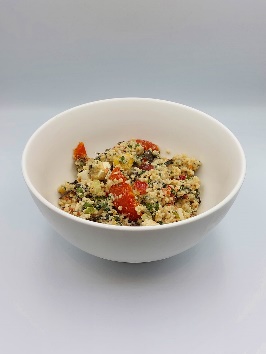 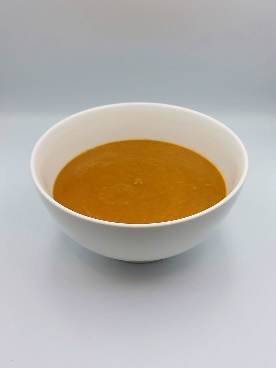 | 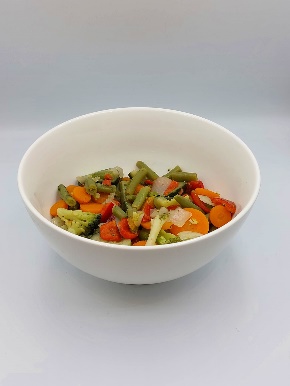 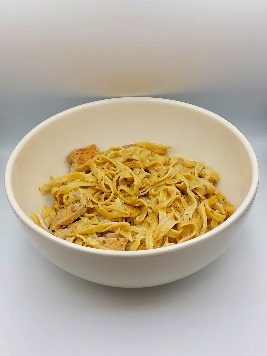 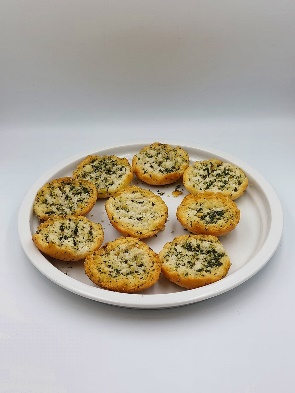 |
|  | - 450g quiche Lorraine (Albert Heijn) - 320g couscous salad with hummus (Albert Heijn) - 455g Harira soup (Albert Heijn) | - 300g Italian vegetable mix (Iglo) - 560g Tagliatelle fungi with 400g fillet pieces (Albert Heijn) - 80g Garlic bread (Pannetteria di Sergio) |
| **Day menu 7** | | |
|  | **Fast** | **Slow** |
| **Breakfast** | 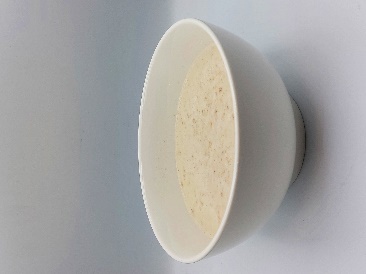 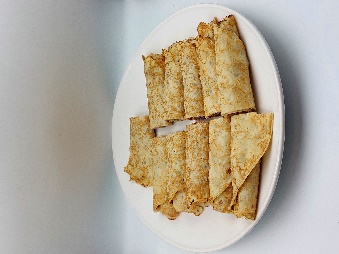 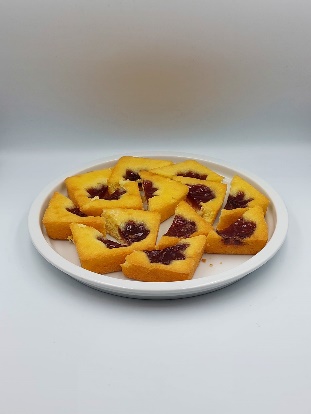 | 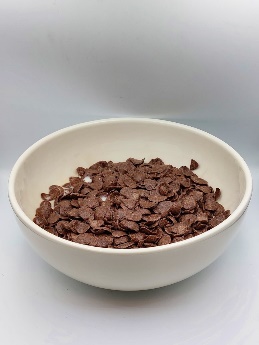 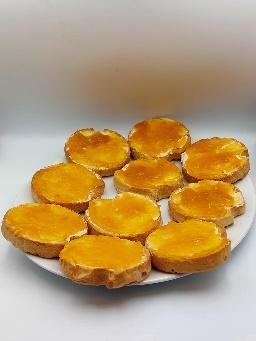 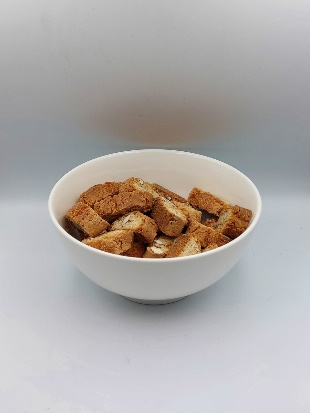 |
|  | - 132g Oats (Quaker) with 360g oatdrink (Oatly) - 371.25g pancake (Jan) with 82.5g apricot jam (Albert Heijn) - 260g Strawberry cakes (Albert Heijn) | - 170g Coco pops (Kellogs) with 510g Oat drink (Alpro) - 96g Rusks (Bolletje) with 50g light margarine (Becel) and 150g apricot jam (Albert Heijn) - 225g cantuccini (Matilde Vicenzi) |
| **Lunch** | - 157g Roasted chicken sandwich (The Bread Office) - 37.5g Liga evergreen (Liga) - 150g White grapes | - 157g Roasted chicken sandwich (The Bread Office) - 37.5g Liga evergreen (Liga) - 150g White grapes |
| **Dinner** | 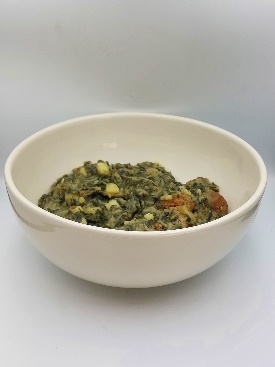 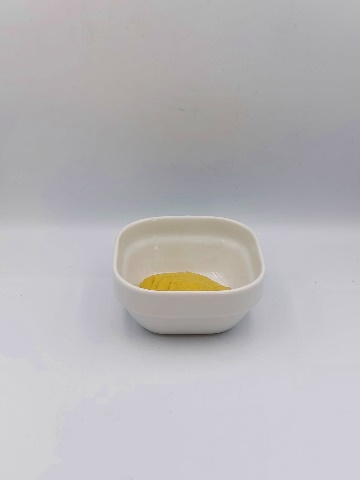 | 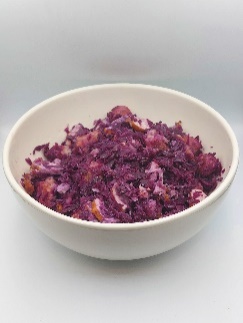 |
|  | - 1160g Andijvie stamppot (Albert Heijn) - 40g Mustard (Albert Heijn) | - 300g small Rösti rounds (Aviko) with 640g red cabbage (Albert Heijn) and 300g smoked chicken pieces (Albert Heijn) |
| **Day menu 8** | | |
|  | **Fast** | **Slow** |
| **Breakfast** | 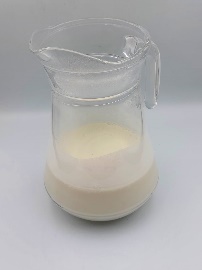 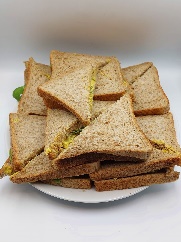 | 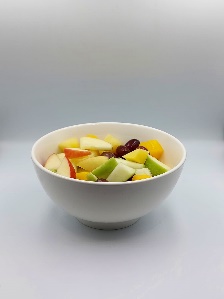 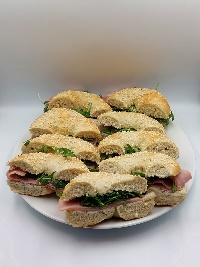 |
|  | - 480g protein drink strawberry raspberry (HIPRO) - 455g casino wholegrain bread with 227.5g chicken curry salad light (Johma) and 32.5g lettuce (Albert Heijn) | - 550g pineapple, mango, apple and grape fruit salad (Albert Heijn) - 382.5g sesame bagels (Albert Heijn) with 225g ham (Albert Heijn) and 45g cream cheese chives light (Castello) and 31.5g arugula (Albert Heijn) |
| **Lunch** | - 157g Bacon egg sandwich (The Bread Office) - 25g B’tween bar peanut butter (Hero) - 160g mandarins | - 157g Bacon egg sandwich (The Bread Office) - 25g B’tween bar peanut butter (Hero) - 160g mandarins |
| **Dinner** | 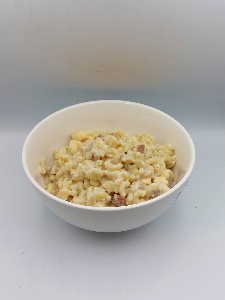 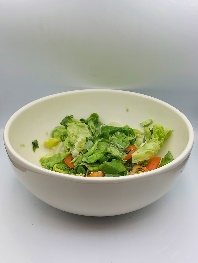 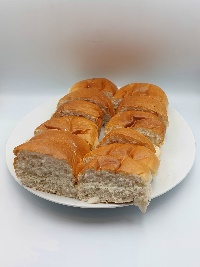 | 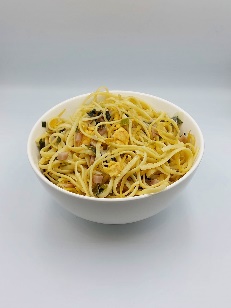 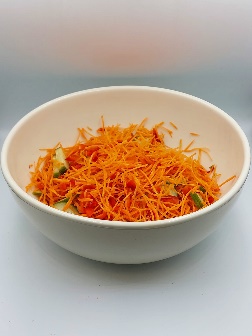 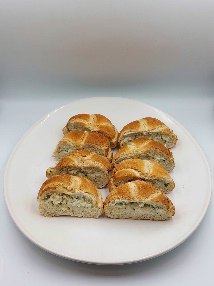 |
|  | - 640g Macaroni Carbonara (Huuskes) - 90g lettuce with 90g tomatoes and 20g natural dressing (Albert Heijn) - 300g white buns (Albert Heijn) with 60g cream cheese herbs (Garlan) | - 620g Bami (Albert Heijn) - 110g red bell pepper, 110g cucumber, 110g carrot julienne (Albert Heijn) and 10g zero calorie dressing (Remia) - 200g Kaiser rolls (Albert Heijn) with 60g cheese with herbs (Heks*n Kaas) |
| **Day menu 9** | | |
|  | **Fast** | **Slow** |
| **Breakfast** | 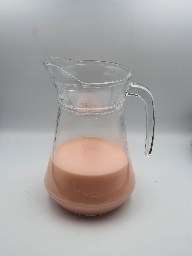 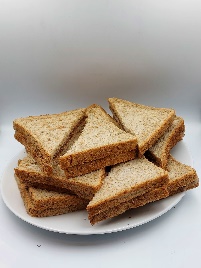 | 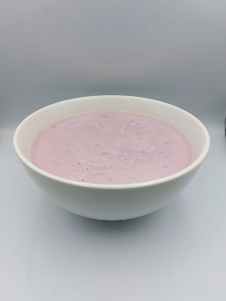 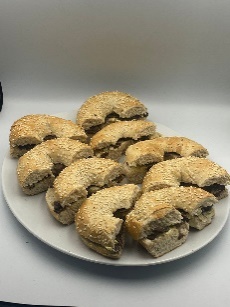 |
|  | - 590g strawberry raspberry protein drink (Melkuni) - 455 casino wholegrain bread with 162.5g Chocolate spread (Nocilla) | - 720g blueberry quark (Optimel) - 382.5g sesame bagels (Albert Heijn) with 22.5g light margarine (Becel) and 90g dark chocolate flakes (de Ruijter) |
| **Lunch** | - 157g Roasted chicken sandwich (The Bread Office) - 37.5g Liga evergreen (Liga) - 150g White grapes | - 157g Roasted chicken sandwich (The Bread Office) - 37.5g Liga evergreen (Liga) - 150g White grapes |
| **Dinner** | 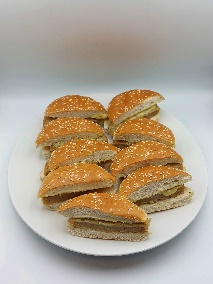 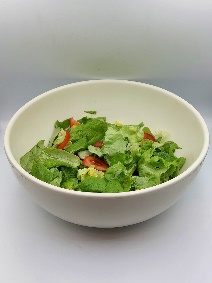 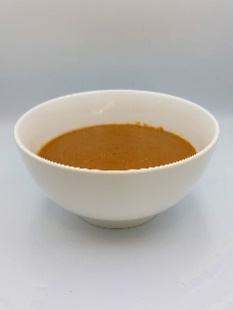 | 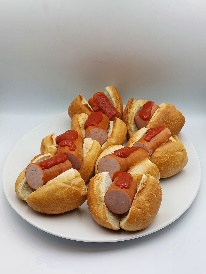 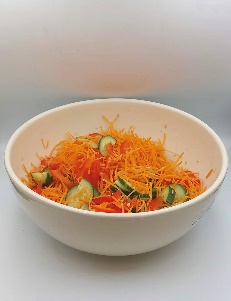 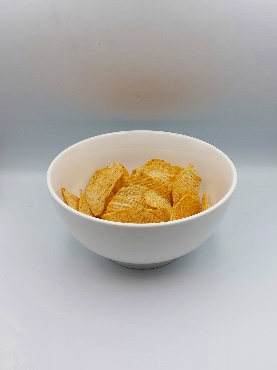 |
|  | - 585g Chicken burger (Mora) - 85g lettuce with 85g tomatoes and 10g zero calorie dressing (Remia) - 450g Harira soup (Albert Heijn) | - 280g white bun (Albert Heijn) with 285g smoked sausage (Unox) and 40g Ketchup zero (Heinz) - 175g red bell pepper, 175g cucumber, 175g carrot julienne (Albert Heijn) and 15g zero calorie dressing (Remia) - 80g Oven baked Mediterranean chips (Lay’s) |

**Supplemental Table 2** Intraclass correlation coefficients (ICC) with 95% confidence intervals for the eating behaviour characteristics obtained with video annotation. An ICC <0.50 indicates poor consistency, 0.50-0.75 indicates moderate consistency, 0.75-0.90 indicates good consistency, and >0.90 indicates excellent consistency [1].

| **Eating behavior characteristics** | **Breakfast** | **Dinner** |
| --- | --- | --- |
| Bites | 0.995 (0.986; 0.999) | 1 (0.998;1) |
| Chews | 0.999 (0.998;1) | 0.999 (0.996;1) |
| Swallows | 0.960 (0.883;0.989) | 0.828 (0.345;0.963) |
| Sips water | 1(1;1) | 0.961 (0.835;0.992) |
| Oro-Sensory Exposure time | 0.999 (0.998;1) | 0.999 (0.964;1) |
| Chewing time | 0.999 (0.998;1) | 0.997 (0.988;0.999) |
| Whole meal duration | 1 (1;1) | 1 (1;1) |
| Water duration | 0.971 (0.912;0.992) | 0.954 (0.808;0.99) |

OSE = oro-sensory exposure

1. Koo, T.K. and M.Y. Li, *A Guideline of Selecting and Reporting Intraclass Correlation Coefficients for Reliability Research.* J Chiropr Med, 2016. **15**(2): p. 155-63.

**Supplemental Table 3** Indicated reasons to stop eating. Data are presented as frequency, n (%). ^1^Other includes: “I got tired of chewing”, Only “unhealthy” foods are left, “I was bored with the texture”, “Enough time has passed since the start of the meal” and “I want to eat the same amount as everyone else”.

| **Reason to stop eating** | **Breakfast** | | **Dinner** | |
| --- | --- | --- | --- | --- |
|  | **Fast** | **Slow** | **Fast** | **Slow** |
| I was full | 130 (54.2%) | 165 (69%) | 148 (67.3%) | 129 (58.9%) |
| The food is no longer appealing to me | 30 (12.5%) | 24 (10%) | 23 (10.5%) | 30 (13.7%) |
| I have eaten the amount that I planned | 27 (11.3%) | 18 (7.5%) | 23 (10.5%) | 11 (5%) |
| I ate the portion that I would normally eat | 19 (7.9%) | 13 (5.4%) | 11 (5%) | 8 (3.7%) |
| I was bored with the flavour | 12 (5%) | 7 (2.9%) | 5 (2.3%) | 8 (3.7%) |
| Other^1^ | 22 (9.2%) | 12 (5%) | 10 (4.5%) | 29 (13.2%) |

**Supplemental Table 4** Average daily dietary intake for each diet, reported as total daily intake and separately for breakfast and dinner meals. Data are presented as mean ± SE. Means in a row without a common superscript letter differ P<0.05 between Fast and Slow diets with Tukey adjustments for multiple comparisons.

|  | **Diet** | | **ANOVA fixed effect** | | |
| --- | --- | --- | --- | --- | --- |
|  | **Fast** | **Slow** | **Diet** | **Day** | **Diet x Day** |
| **Diet** |  |  |  |  |  |
| Cumulative food intake (g) | 16789 ± 436 | 15733 ± 436 | <0.001 | NA | NA |
| Cumulative energy intake (kcal) | 25196 ± 624 | 24798 ± 624 | 0.08 | NA | NA |
| **Day** |  |  |  |  |  |
| Daily Food intake (g/day) | 1526 ± 64^a^ | 1430 ± 65^b^ | <0.001 | <0.001 | 0.35 |
| Daily Energy intake (kcal/day) | 2291 ± 90 | 2254 ± 9 | 0.28 | <0.001 | 0.52 |
| Fat intake (g/day) | 88 ± 4 | 78.2 ± 4 | <0.001 | <0.001 | 0.6 |
| Carbohydrates (g/day) | 275 ± 10 | 274 ± 10 | 0.68 | <0.001 | <0.001 |
| Sugar intake (g/day) | 88 ± 3 | 90 ± 3 | 0.32 | <0.001 | 0.003 |
| Fiber intake (g/day) | 28 ± 1 | 28 ± 1 | 0.95 | <0.001 | 0.008 |
| Protein intake (g/day) | 85 ± 4 | 83 ± 4 | 0.4 | <0.001 | 0.006 |
| Sodium intake (g/day) | 10 ± 0.5 | 9 ± 0.5 | 0.006 | <0.001 | 0.005 |
| **Breakfast** |  |  |  |  |  |
| Food intake (g) | 449 ± 29^a^ | 483 ± 29^b^ | 0.03 | <0.001 | 0.21 |
| Energy intake (kcal) | 700 ± 40^a^ | 748 ± 40^b^ | 0.02 | <0.001 | 0.05 |
| Fat intake (g) | 23 ±1 | 21 ± 1 | 0.05 | <0.001 | 0.005 |
| Carbohydrates (g) | 94 ± 6^a^ | 107 ± 6^b^ | <0.001 | <0.001 | 0.02 |
| Sugar intake (g) | 38 ± 3^a^ | 45 ± 3^b^ | 0.005 | <0.001 | 0.03 |
| Fiber intake (g) | 8 ± 1^a^ | 9 ± 1^b^ | 0.03 | 0.04 | <0.001 |
| Protein intake (g) | 25 ± 2 | 25 ± 2 | 0.52 | <0.001 | 0.66 |
| Sodium intake (g) | 1.8 ± 0.1 | 1.8 ± 0.1 | 0.72 | <0.001 | 0.56 |
| **Dinner** |  |  |  |  |  |
| Food intake (g) | 737 ± 42^a^ | 607 ± 42^b^ | <0.001 | <0.001 | 0.29 |
| Energy intake (kcal) | 991 ± 56^a^ | 907± 56^b^ | 0.002 | <0.001 | 0.85 |
| Fat intake (g) | 44 ± 2^a^ | 36 ± 2^b^ | <0.001 | <0.001 | 0.78 |
| Carbohydrates (g) | 105 ± 6^a^ | 91 ± 6^b^ | <0.001 | <0.001 | <0.001 |
| Sugar intake (g) | 20 ± 1^a^ | 15 ± 1^b^ | <0.001 | <0.001 | <0.001 |
| Fiber intake (g) | 15 ± 1 | 14 ± 1 | 0.18 | <0.001 | 0.05 |
| Protein intake (g) | 36 ± 2 | 36 ± 2 | 0.56 | <0.001 | <0.001 |
| Sodium intake (g) | 5.9 ± 0.4 ^a^ | 5.4 ± 0.4 ^b^ | 0.01 | 0.007 | 0.003 |

|  | **Fast** | | | **Slow** | | | **ANOVA fixed effect P-value** | | |
| --- | --- | --- | --- | --- | --- | --- | --- | --- | --- |
|  | **Day 1** | **Day 5** | **Day 12** | **Day 1** | **Day 5** | **Day 12** | **Diet** | **Day** | **Diet x Day** |
| **Body weight (kg)** | 73.7 ± 2.1 | 73.3 + 2.1 | 73.0 ± 2.1 | 73.5 ± 2.1 | 73.3 ± 2.1 | 72.8 ± 2.1 | 0.32 | 0.006 | 0.82 |
| **Waist-to-hip ratio** | 0.76 ± 0.02 |  | 0.78 ± 0.02 | 0.77 ± 0.02 |  | 0.75 ± 0.02 | 0.59 | 0.88 | 0.18 |
| **FM (kg)** | 23.0 ± 2.3 |  | 22.5 ± 2.3 | 22.8 ± 2.3 |  | 22.3 ± 2.3 | 0.93 | 0.81 | 1 |
| **FFM (kg)** | 43.1 ± 1.9 |  | 43.4 ± 1.9 | 43.2 ± 1.9 |  | 43.6 ± 1.9 | 0.95 | 0.86 | 0.99 |

**Supplemental Table 5** Body weight measured at the start, midpoint and end of the fast and slow diets. Waist-to-hip ratio measured at the start and end of the fast and slow diets. Fat mass and fat free mass derived from bio-electrical impedance analysis at the start and end of the fast and slow diets. Data are presented as mean ± SE.

FM= Fat mass

FFM = Fat Free mass
